# Effects of simulation in improving the self-confidence of student nurses in clinical practice: a systematic review

**DOI:** 10.1186/s12909-023-04793-1

**Published:** 2023-10-30

**Authors:** Nojoud Alrashidi, Eddieson Pasay an, Maha Sanat Alrashedi, Aidah Sanad Alqarni, Ferdinand Gonzales, Enas Mohammed Bassuni, Petelyne Pangket, Lorraine Estadilla, Lizy Sonia Benjamin, Kawther Elthayeb Ahmed

**Affiliations:** 1 College of Nursing, University of Hail, Hail City, Saudi Arabia; 2 College of Nursing, King Khalid University, Abha, Saudi Arabia

**Keywords:** Simulation, Self-confidence, Student nurses, Clinical practice

## Abstract

**Background:**

Considering the positive influence of simulation from previous literature may encourage educators to regard it as a valuable teaching strategy in nursing schools. This literature review aims to investigate whether the use of simulation improves undergraduate nurses’ self-confidence during clinical practice.

**Methodology:**

This study employed a literature-based design. Five academic databases, including CINAHL, EBSCO, ProQuest, PubMed and Medline, were utilised to extract relevant studies using appropriate keywords and Boolean operators. Studies published in the last 15 years (2005–2020) were included in the search. Studies were retrieved using the Exclusion and Inclusion criteria. The Critical Appraisal Skills Programme (CASP) was used to critically appraise the studies.

**Results:**

A total of 15 primary research studies were extracted for review. Four major themes emerged from the review: Improved self-confidence to carry out clinical tasks, Increased ability to work in teams, Improved self-confidence to perform community work and Improved self-confidence in communicating with patients and team members.

**Conclusion:**

Clinical simulation is a useful tool in increasing the self-confidence of nursing students to perform clinical tasks, make clinical judgements, communicate with patients and team members and improve their teamwork. To improve the quality of care received by patients, it is recommended that clinical simulations be integrated into the nursing curriculum in Saudi Arabia. Increasing the confidence of students has been shown to be associated with greater confidence in performing clinical tasks.

## Introduction

Nurse educators view repetitive simulation as a beneficial teaching strategy for supporting students’ learning objectives [1]. It provides an experiential environment that eases the feeling of pressure while on placement [2]. As an innovative method of teaching using simulation-based clinical education, nursing students are provided the opportunity to hone their clinical and decision-making abilities through a variety of real-life situational encounters without jeopardising the patient’s safety [3]. Guerrero and colleagues [4] defined simulation as an educational method for students to gain and enhance professional competence, for example, uses simulation, a technology that simulates real-life scenarios. Simulation is an extremely effective tool for creating a number of different learning outcomes [5]. Moreover, it could provide an environment where learners can develop skills without jeopardising the safety of others. A previous study maintained that students who participated in simulations showed excellent attitudes towards learning and had increased competence and confidence [6]. In other words, students finish simulations effectively, they gain confidence in their ability to apply the same abilities in the real world.

In simulation, the concept of self-confidence is the degree of assurance and trust that nursing students have in their capacity to carry out the necessary duties and hone the necessary abilities in a simulated clinical situation. Self-confidence is a key component of nursing education and practice, and it is positively connected with student satisfaction and their capacity for critical thought. Through consistent practice, constructive criticism, and debriefing sessions, self-confidence can be built [7]. To develop self-confidence in simulation, a safe and encouraging learning environment is essential. For students to take risks and try new things, they must feel comfortable and supported [8]. In order to do this, the simulation environment needs to foster an atmosphere of mutual respect and cooperation [9]. When students perform well, instructors can provide them encouragement and good feedback, which can boost their confidence.

In nursing education, simulation is crucial for the formation of clinical judgment [10, 11]. It is a cutting-edge method of instruction that aim to replicate key features of clinical cases [12] with effectiveness and learning impact proven in areas like decision-making and clinical reasoning, simulation is acknowledged as a valuable learning tool in nursing education [13]. A perfect environment for honing clinical judgment skills is provided by recent advancements in high-fidelity simulation [10]. Students benefit from simulation by getting hands-on experience, which is crucial for developing clinical judgment, and by getting immediate feedback on how they’re doing [10]. Even while there is no doubt that clinical judgment is tied to real-world experience, students benefit from clear objectives and honest evaluations of their performance. The use of simulation in professions that involves protecting the lives of people could provide an environment where professionals learn skills without compromising the safety of individuals. Al-Elq [14] added that, in contrast to learning skills by performing procedures on patients, simulation allows nursing students to acquire clinical skills through practice with a patient model. Simulation tools would serve as an optimal alternative to actual patients in healthcare settings. Overall, the aforementioned suggest that using simulation as a teaching tool is effective in helping nursing students strengthen their clinical judgment. To assist in the development of the knowledge and skills required for successful nursing, educators should think about introducing simulation into their courses.

To support the use of simulation in nursing, a framework for creating and executing simulation-based education programs that are efficient, successful, and evidence-based is provided by the NLN Jeffries Simulation Theory [15]. The idea places a strong emphasis on the value of context, background, design, instructional practices, simulation experience, and outcomes in simulation-based learning. Indeed, it is an effective tool for researchers and educators who are interested in developing and examining simulation-based nursing education programs. Simulation is an evidence-based, learner-centered, collaborative learning and improvement technique [16]. The best available evidence should be used to inform the design and implementation of simulation in order to fulfil the demands of the students. Building teamwork and communication skills, improving patient outcomes, and enhancing learning are all possible using simulation.

While there is a paucity of literature on the use of simulation in nursing curricula in Saudi Arabia, consideration should also be made that simulation is an emerging tool in nursing education in Saudi Arabia; hence, there is a lack of studies investigating the influence of this type of teaching method on student learning in the country. However, this dearth of literature does not imply that nursing curriculum developers overlooked the potential of simulation as a means of improving clinical competence, teamwork and other needed skills. Notably, this review is important because it assists nursing curriculum developers in making decisions on the use and application of simulation in nursing education. Determining whether the impact of simulation extends beyond the student years would help improve the current knowledge of its effectiveness. Analysing and evaluating studies helps nursing educators take advantage of the potential uses of simulation in developing nurses who are clinically competent. Consequently, this study aims to find evidence from the literature on whether the use of simulation improves undergraduate nurses’ self-confidence in performing their clinical tasks. The goal of this review was to answer the research question—Does simulation increase the self-confidence of student nurses? This can be translated into PICO as: P: Student nurses; I: Simulation; C: No simulation; O: Increase in self-confidence.

## Materials and methods

### Search strategy

Academic databases, including CINAHL, PubMed, Ebsco and ProQuest, were used to identify and access recent and relevant published studies on the use of simulation amongst nursing students and whether simulation could increase the confidence of undergraduate nursing students during their clinical practice. The Boolean operator AND was used to identify studies that contained the keywords of the search terms. This was to ensure that the retrieved studies would contribute to new knowledge or validate what has been previously known about clinical simulation. The keywords entered into these databases included simulation AND nursing students AND confidence, simulation AND nursing AND confidence, nursing AND simulation, and simulation AND confidence. The Boolean operator ‘AND’ ensured that all keywords entered in the database would be reflected in the studies retrieved for the review.

### Inclusion and exclusion criteria

The inclusion criteria of this systematic review includes (1) Studies published between 2005 and 2020 because the use of clinical simulations in the nursing curriculum have increased in the last 15 years, (2) studies published in English, (3) studies that are in full text, and (4) primary research studies to ensure that first-hand evidence was analysed and applied to nursing curriculum development. Exclusion criteria are as follows (1) studies that are not peer-reviewed, and (2) studies that do not measure the outcomes of interest.

### Critiquing framework

The studies were critiqued using the Critical Appraisal Skills Programme (CASP) [17, 18] tools for critiquing quantitative and qualitative studies. These tools were chosen because of their credibility in critiquing studies. The critiquing framework for quantitative studies developed by Long et al. [12] was used to determine the quality of the quasi-experimental and survey studies included in the present review. The CASP tool was also used to examine whether the results could be applied to the local population and whether all clinically important outcomes were taken into consideration.

The CASP [17] tool for qualitative studies was also used. It begins with three screening questions that review the results, their validity and whether those results can be applied to local populations. This tool contains 10 questions. It starts with a question on whether the aims and objectives are clearly stated and investigates whether a qualitative methodology was appropriate to answer the aims and objectives. Then, it examines the appropriateness of the research design to address the research aims and objectives.

## Results

### Search results

A total of 15 studies that met the inclusion criteria set in this study were retrieved from academic databases. A detailed review of the studies is provided in Table 1. We used three phases in the conduct of this review.

**Table 1:** Summary of the included articles using Inclusion/Exclusion criteria

S/ No	Author/Year	Country	Aim	Type of study	Critical results
1	Bambini et al. (2009) Outcomes of clinical simulation for novice nursing students: communication, confidence, clinical judgement/ 2009	USA	Investigation whether clinical simulation was associated with increased self-efficacy in nursing students	Quasi- experimental study	Novice nursing students who participated in clinical simulation exercises demonstrated significant improvement in their communication skills,confidence and clinical judgement.
2	Blum et al. (2010) High-fidelity nursing simulation: impact on student self-confidence and clinical competence/ 2010	USA	Measurement of the self- confidence and competence of entry level nursing students following a simulation-enhance laboratory	Quasi- experimental study design	Participation in high-fidelity nursing simulation (HFS) was associated with a significant increase in student self-confidence and clinical competence
3	Tawalbeh and Tubaishat (2014) Effect of simulation on knowledge of advanced cardiac life support, knowledge retention, and confidence of nursing students in Jordan/ 2014	Jordan	The study examined the effect of simulation on nursing students’ knowledge of advanced cardiac life support (ACLS), confidence in applying ACLS skills and knowledge retention	Experimental, randomised controlled (pre- test-post-test) design	Simulation was found to be more effective than traditional teaching methods in improving nursing students’ knowledge of advanced cardiac life support (ACLS), knowledge retention, and confidence.
4	Richards et al. (2010) Public health nursing student home visit preparation: the role of simulation in increasing confidence/2010	USA	Evaluation of the role of simulation in preparing senior nursing students for their first home visit and to determine comfort and confidence levels of these students	Exploratory study; pre-test post-test design was used.	Simulation was found to be an effective method for increasing the confidence of public health nursing students in preparing for home visits.
5	Smith and Roehrs (2009) High-fidelity simulation: factors correlated with nursing student satisfaction and self-confidence/ 2009	USA	Investigation of the factors correlated with nursing student satisfaction and self- confidence	Descriptive, correlational study	Simulation design factors, such as the use of realistic objectives and problem-solving activities, were correlated with nursing student satisfaction and self-confidence.
6	Thomas and Mackay (2012) Influence of a clinical simulation elective on baccalaureate nursing student clinical confidence. 2012	USA	Investigation on whether high- fidelity simulation course significantly changes students’ level of confidence, when compared with a traditional clinical experience	Quasi- experimental; use of pre-test- post-test design	The participation in a clinical simulation elective was associated with a significant increase in baccalaureate nursing student clinical confidence.
7	Valizadeh et al. (2013) The effect of simulation teaching on baccalaureate nursing students’ self- confidence related to peripheral venous catheterization in children: A randomized trial	Iran	Investigation on whether simulation teaching method could increase self-confidence of students in performing peripheral venous catheterization in paediatric patients	Randomised controlled trial	Simulation teaching was found to be effective in improving baccalaureate nursing students’ self-confidence related to peripheral venous catheterization (PVC) in children.
8	Franklin et al. (2014) Psychometric testing on the NLN student satisfaction and self- confidence in learning, simulation design scale, and educational practices questionnaire using a sample of pre- licensure novice nurses	USA	This study investigated the reliability of the Student Satisfaction and Self- Confidence in Learning Scale, Simulation Design Scale, and Educational Practices Questionnaire	Quantitative- item analysis, confirmatory and exploratory factor analyses in randomly-split subsamples, concordant and discordant validity and internal consistency	Student’s satisfaction with their learning experiences and their confidence in their ability to learn
9	Ballangrud et al. (2013) Intensive care unit nurses’ evaluation of simulation used for team training	Norway	This study investigated intensive care nurses’ evaluations of simulation used for team training	Questionnaire Evaluation design	Simulation used for team training was found to be an effective way to improve intensive care unit (ICU) nurses’ knowledge, skills, and confidence in managing critical events.
10	Kjellin et al. (2014) Hybrid simulation: Bringing motivation to the art of teamwork training in the operating room	Norway	This study investigated situational motivation and self- efficacy of healthcare practitioners receiving hybrid simulation training in the operating room	Quantitative- questionnaire was used to test confidence and skills of the trainees before and after the training	Hybrid simulation group had significantly higher scores on the TeamSTEPPS Team Performance Assessment Tool and the Communication and Teamwork Skills Scale than the control group. The hybrid simulation group also reported higher levels of satisfaction with the training experience and higher levels of confidence in their teamwork and communication skills.
11	Hart et al. (2014) Effectiveness of a structured curriculum focused on recognition and response to acute patient deterioration in an undergraduate BSN program	USA	The study evaluated the effectiveness of a structured education curriculum with simulation training for undergraduate nursing students	Qualitative and quasi- experimental	Structured curriculum focused on recognition and response to acute patient deterioration in an undergraduate BSN program can significantly improve students’ knowledge, self-confidence, and perceptions of teamwork.
12	Clay-Williams et al. (2013) Classroom and simulation team training: a randomized controlled trial	Sydney, Australia	This study investigated whether simulation-based crew resource management training and classroom training interventions improved the behaviours and teamwork attitudes of participants	Randomized controlled trial	Classroom-based team training alone resulted in improvements in participant knowledge and observed teamwork behavior, but simulation-based training did not provide any additional benefits.
13	Liaw et al. (2014) An interprofessional communication training using simulation to enhance safe care for a deteriorating patient	Singapore	This study aimed to evaluate the Sim-IPE programme in improving communication skills of students caring for patients with physiological deterioration	Quasi- experimental	Interprofessional communication training using simulation can significantly improve healthcare professionals’ confidence in communicating with each other, managing deteriorating patients, and working as a team.
14	Mager and Campbell (2013) Home care simulation for student nurses: medication management in the	USA	Examined the effects of HC simulation model on the knowledge and confidence in managing medications	Quasi- experimental	Home care simulation can significantly improve student nurses’ knowledge and confidence in managing medications in the home setting.
15	Yang et al. (2012) The effect of clinical experience, judgment task difficulty and time pressure on nurse’s confidence calibration in a high fidelity clinical simulation	USA	Examination of nurses’ calibration of confidence with judgement accuracy for critical event risk assessment judgements. The study also explored the effects of clinical experience, task difficulty and time pressure on the relationship between confidence and accuracy	Quasi- experimental	Nurses were overall poorly calibrated, with both student and experienced nurses being overconfident in their judgments. The clinical experience did not improve calibration where experienced nurses were even more overconfident than student nurses.

In the first phase, we identify all of the relevant literature on the topic of interest. This is done by searching electronic databases and other sources of information. The search results are then screened to identify the articles that are most relevant to the review question. This screening process is typically done by reviewing the titles and abstracts of the articles. The second phase is the eligibility assessment where once the relevant articles have been identified, we assessed for their eligibility. This involves reviewing the full text of each article to determine whether it meets the inclusion and exclusion criteria of the review. The inclusion criteria are the characteristics that the articles must have in order to be included in the review. The exclusion criteria are the characteristics that disqualify an article from being included in the review. The third phase is the data extraction and synthesis. Once the eligible articles have been identified, the data is extracted from each article. This data is then synthesized to provide a comprehensive overview of the evidence on the topic of interest. The synthesis involved summarizing the findings of the individual studies, comparing the findings of different studies, and identifying any patterns or trends in the data.

Duplicates were eliminated and discrepancies were resolved by consensus to address issues of rigor and bias and this ensures that each article is only counted once. We did not register our review in the International Prospective Register of Systematic Reviews (PROSPERO) because it did not meet the qualifying requirements.

#### Study characteristics

Of the 15 studies, eight were conducted using quasi-experimental designs [19–27]. The participants in the studies are nursing students, nurses, and other healthcare professionals where the interventions in the studies all involve clinical simulation. While the outcomes of the studies vary, they all suggest that clinical simulation can have a positive impact on healthcare professionals’ knowledge, skills, and confidence and provided strong evidence that clinical simulation is an effective teaching and training method for healthcare professionals. In addition, three studies [28–30] were conducted using randomized control where they have provided strong evidence that clinical simulation is an effective teaching and training method for healthcare professionals. Four studies conducted employing descriptive correlation and evaluation analysis [31–33] also provide strong evidence that simulation is an effective teaching and training method for healthcare professionals. These studies also provide important information about the factors that contribute to student satisfaction and confidence in simulation training.

**Fig. 1:**
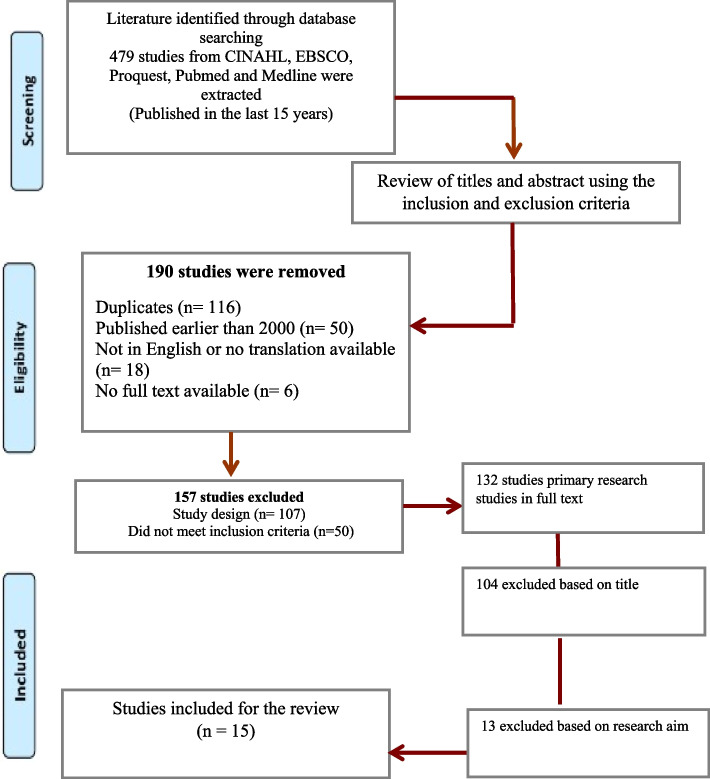
Retrieval of studies from academic databases

The succeeding figure summarises the search and retrieval of the 15 studies (See Fig. 1). These findings are arranged according to the main themes that were common amongst them. Four major themes emerged in the review: Improved self-confidence to carry out clinical tasks, Increased ability to work in teams, Improved self-confidence to perform community work and Improved self-confidence in communicating with patients and team members. Each of these themes will be discussed along with the studies that support them.

### Thematic analysis

**Fig. 2:**
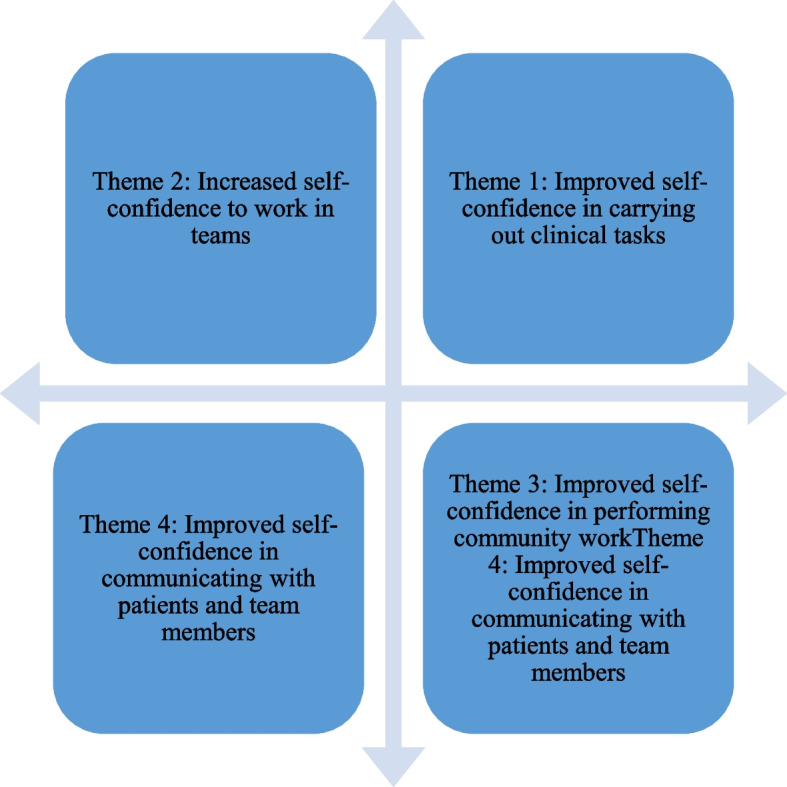
Integrated main themes

Thematic analysis is an identification of the major themes emerging from the critical appraisal and review of individual studies [17]. The succeeding section presents the four major themes that emerged in the study. It also looks at the studies that support each of these themes. Figure 2 presents the integrated main themes of the review.

### Theme 1: improved self-confidence in carrying out clinical tasks

Most of the studies [19–33] suggested that clinical simulation improves the self-confidence of student nurses in carrying out clinical tasks. They found self-confidence to be associated with nurses making more accurate clinical judgements during critical situations. While experienced registered nurses were found to be overconfident, it was suggested that student nurses were under confident in making clinical judgements. However, when student nurses received clinical simulation training, their level of self-confidence improved. This is essential not only in making clinical judgements, but also when performing important procedures for patients. Since these nurses have practised virtually or on patient models, they perceive that they can perform similar procedures with real patients.

Clinical simulation provides a relatively safe environment, since student nurses initially perform procedures on patient models and not on live patients. This provides them with time to think through a task and perform procedures they have learned in the classroom and during simulations. Since they receive feedback if they perform tasks erroneously, they immediately become aware of how to perform the procedure correctly. This could provide them with the needed self-confidence and assurance that they can perform correct procedures on real patients.

### Theme 2: increased self-confidence to work in teams

Some of the studies [22, 26, 29] in this review supported the theme that engagement in clinical simulation training increases the self-confidence of student nurses to work in teams. This theme is also related to the first theme, in which practicing their skills on virtual patients helped improve their confidence in performing the same procedures on real patients. Since most nurses are involved in teamwork, the knowledge and skills they gained in performing tasks could have improved their self-confidence to share their learning with other team members. Since effective teamwork is essential in improving the health outcomes of patients, this theme has important implications for nursing practice. The Ministry of Health in Saudi Arabia strongly supports teamwork between nurses and other healthcare practitioners. Developing effective teamwork skills early in the nursing undergraduate years will prepare student nurses to be effective team members in their post-registration years. Nurse educators should consider the integration of this tool in the nursing curriculum in Saudi Arabia because clinical simulation is associated with increased confidence in participating in teams.

### Theme 3: improved self-confidence in performing community work

Three of the studies [22, 25, 33] suggested that clinical simulation allowed student nurses to gain the self-confidence to perform community work. These findings have important implications in nursing practice, since the work of nurses is not limited to clinical settings and it involves community work as well. Increasing the self-confidence of nurses to perform community work could help improve the quality of care. Moreover, these nurses are now empowered to increase the self-efficacy of patients during their community work.

### Theme 4: improved self-confidence in communicating with patients and team members

Five studies [19, 22, 26, 29, 32] reflected the theme that attendance in clinical simulations is associated with improved self-confidence in communicating with patients and team members. Communication [34] plays an integral role in improving the quality of care received by patients. Because team members are able to articulate their feelings, perceptions and ideas with the team, positive communication influences the quality of the healthcare that they provide. Gaining self-confidence to contribute to the team’s goals and objectives would help nurses set goals that could help improve the quality of life of patients. In the present review, it is suggested that participation in clinical simulation classes will allow student nurses to gain the confidence to communicate with patients and other team members. When sustained over time, this confidence will allow student nurses to develop their communication skills and interact effectively with patients and colleagues.

## Discussion

This review aims to find evidence from the literature on whether the use of simulation improves undergraduate nurses’ self-confidence in performing their clinical tasks. The primary goal of simulation training is to ensure that student nurses acquire the requisite skills to perform clinical procedures, participate in collaborative work with other healthcare practitioners and enhance safety during the actual care of patients [14]. One way to achieve quality care is to ensure that student and registered nurses are confident in carrying out clinical tasks and procedures and in working effectively with patients and their family members in community and other healthcare settings. The capability of student nurses to be confident in making clinical judgements and performing clinical procedures is a critical part of effective and safe healthcare [34]. The results of the present review strongly suggest that attendance in simulation training improves the ability of student nurses to conduct Advanced Cardiovascular Life Support (ACLS) and perform procedures such as peripheral venous catheterisation. Both procedures require repeated practice before student nurses are regarded as competent by their mentors [34]. Apart from refining the skills of the student nurses, simulation training additionally improved the teamwork of the students with other healthcare professionals. This is an important trait since nurses are encouraged to lead healthcare and collaborate with other healthcare practitioners when creating healthcare innovations or planning the care and management of the healthcare conditions of patients [35].

Developing the self-confidence of student nurses at an early stage might improve their ability to provide safe and effective healthcare. Most of the studies included in this review showed a strong association between simulation training and an increase in the reported self-confidence of student nurses. Under confidence to perform clinical tasks or make clinical judgements could compromise patient safety [35]. Gao et al. [36] argued that both overconfidence and under confidence in making judgements during critical events may result in inappropriate or delayed interventions. Nurses might experience being over or under confident in making decisions. Gao et al. [36] also reiterate that the overconfidence of nurses in making decisions makes them prone to making incorrect decisions because they are less likely to actively seek the help of others. This increases their likelihood of making erroneous clinical judgements, and it compromises patient safety. Similarly, nurses being under confident might lead to delays in making clinical judgments or interventions.

Increasing the confidence of student nurses is crucial to helping them make appropriate clinical judgements and perform clinical procedures. Goldhill [37] suggested that confidence in making clinical judgements and performing procedures appears to be linked to experience and difficulty in making judgements. The findings of Yang et al. [24] maintained that when compared to registered nurses, student nurses tended to report being under confident when making clinical judgements, even following simulation training. This contrasts with the rest of the studies, which argued that student nurses were generally confident in performing clinical procedures, communicating interprofessionally and participating in teamwork. However, Yang et al. [24] compared the findings of student nurses with those of registered nurses. This marked difference could have contributed to the observation that student nurses were generally less confident in making clinical judgements.

These findings have an important connection with the results of the other studies reviewed in the present study. It should be noted that all the interventions were only done for a few sessions, with one session only lasting three hours [22]. This promotes safe and effective care and ensures that students learn in a safe environment without the risk of harming patients [35]. This also suggests that, even with limited exposure time to simulation training, this significantly increases the self-confidence of student nurses. In addition to clinical simulation, the continuous experiences of the nurses as they progress from being students to registered nurses would add to their confidence in clinical judgement and procedures. The focus of this review is limited to investigating the relationship between clinical simulation and nurses’ confidence in performing procedures or clinical tasks. However, Yang et al. [24] suggested that confidence in clinical judgement might be developed after several experiences with an acute or critical event and not exclusively through simulations in classroom settings.

Crookes et al. [38] stated that an appropriate level of confidence reflects an individual’s clinical experience, and it also marks one’s competency. Therefore, clinical experience is suggested to be a factor in developing the confidence of nurses when making clinical judgements. The present review shows that even amongst student nurses, confidence in performing clinical tasks and making clinical decisions could be increased after simulation training. Nonetheless, the findings of this review should still be taken with caution. All the studies had a relatively small sample size, which limits their application to a larger and more heterogeneous population [39]. The outcomes measured in the studies were also different. However, all the studies showed that the self-confidence to perform clinical tasks, make clinical judgements, participate in teams and communicate with patients was evident amongst the student nurses following attendance in clinical simulation sessions. It is noteworthy that although most of these sessions only lasted for a few hours, they had an impact on the knowledge and self-confidence of the participants. This suggests that clinical simulation could be considered an important tool in helping student nurses gain self-confidence.

Hart et al. [26] explained that it is essential for nurse educators to use innovations during teaching to address the gap between theory and practice in nursing. The use of innovations in clinical simulation in teaching students how to assess a patient, communicate with them, perform clinical procedures and make clinical judgements has already been used for several years in numerous countries throughout the world. However, its application in Saudi Arabia has yet to be maximised, especially in the nursing curriculum. As shown in the studies reviewed for this investigation, there is strong evidence that clinical simulation is effective in helping student nurses gain confidence. Since all the studies included student nurses as part of the sample population, it should be noted that this self-confidence was further enhanced during post-registration [35]. Intensive care nurses, for example, expressed high satisfaction with simulation-based learning. Further, it also enhanced their self-confidence in performing critical procedures for their patients.

Students learn differently, and their prior learning could influence how they accept clinical simulation as a teaching strategy. In Saudi Arabia, for example, student nurses learn through theory and practice. The latter is accomplished during placements in different clinical settings and is supervised by senior nurses when performing clinical tasks [9], which provides them an opportunity to enhance their clinical skills. However, this also increases the risk of compromising patient safety, especially if students are not confident in performing clinical tasks [35]. At present, there are no established guidelines on the number of hours that could be used for clinical simulation in Saudi Arabia’s undergraduate nursing curriculum. This contrasts with the UK [40], where educators are allowed to incorporate 300 h of simulation as part of teaching actual clinical skills to nursing students.

The present literature-based study could provide a background on how clinical simulation improves patient safety by increasing the self-confidence of student nurses. This self-confidence could extend until post-registration, and it would enable the student nurses to be better prepared in their future roles. The wide application of clinical simulation in clinical practice may also promote teamwork. Nurses frequently work in teams, and developing them to be confident team contributors ensures that they become active participants during multidisciplinary teamwork [35].

As previously noted, each student learns differently. Introducing a simulation-based curriculum should be done only after students have sufficient knowledge and experience to provide meaning to the instructions given during simulation. Jarvis and Rivers [41] utilised constructivist theory as the basis for developing critical thinking skills amongst students who were enrolled in a simulation-based curriculum. Learners are identified as visual, audio read/write and kinaesthetic learners (VARK) [34]. Clinical simulation could benefit kinaesthetic learners the most since it allows them to practise their skills on virtual or model patients. Visual and audio learners could benefit from clinical simulation since they can hear and see clinical procedures. In contrast, read/write learners may benefit from this type of learning as they reflect on the learning and write down their reflections or read about the clinical task before their participation in clinical simulation training or sessions. The findings of the studies also suggested the impact of clinical simulation on the future practice of nursing students. As students gain self-confidence, their ability to provide quality care also improves.

The introduction of a simulation-based nursing curriculum in Saudi Arabia was preceded by the introduction of a similar curriculum in the medical field [14]. Integrating simulation-based learning in the nursing curriculum in Saudi Arabia might increase the self-confidence of nursing students. Notably, the present review showed that simulation is strongly associated with improvements in self-confidence when communicating with patients and colleagues. McCabe [42] argued that effective communication is possible when nurses have the confidence to communicate effectively with patients. Increasing the competence of nurses has been noted as an effective means of increasing their self-confidence [42]. As demonstrated in this review, the introduction of clinical simulation and the actual practice of students on what they learned on model or virtual patients could have resulted in perceived competence in clinical skills. This could have allowed nurse students to perceive that they are not only confident in their clinical skills, but also in communicating what they have learned with patients and their colleagues. The studies included in this review strongly suggested that the quality of care is enhanced when the nurse-patient relationship is enhanced. Since effective communication underpins the nurse-patient relationship, increasing the self-confidence of nurses to communicate with their patients is one way of improving patient care.

### Limitations of the study

Despite limitations, it is noteworthy that all the studies were able to demonstrate that, even with short training sessions on clinical simulation, its impact on the self-reported self-confidence of the students was significant. There were noteworthy positive changes following the learning intervention. Although most of these significant changes were seen in pre-and post-test evaluations, and not through comparisons with a control group, the findings were able to establish that clinical simulations could improve the self-confidence of the student nurses. Conversely, we did not include grey literature, which may have had a possible benefit on the results. Also, the majority of the studies originated from Western countries; thus, extrapolation of these results to Eastern populations is questionable. Meanwhile, many of the studies suffered from significant sources of bias, which should be taken into consideration in future interrogations. On many occasions, the effect was assessed by very few studies; thus, the evidence to support it was low, and the small sample sizes further acted as a limitation on the representativeness of the findings to the population of student nurses.

### Implications to nursing/medical education and practice

This review has significant ramifications for nursing and medical practice. The self-confidence of students needs to grow if they are to develop post-registration skills and be confident in handling their duties. As this review has demonstrated, developing competence in carrying out clinical tasks or procedures is related to one’s level of confidence. According to research, clinical simulation training can assist students in becoming proficient in their chosen clinical procedures, clinical judgement and interpersonal communication. The students’ performance in class and the standard of care given to patients may increase once they believe that they are now capable of communicating and carrying out activities. Finding solutions to improve quality care is crucial for nurse educators because the goal of healthcare is to always deliver patient-centred care.

According to this review, clinical simulation is a technique that can both boost students’ self-confidence and enhance the standard of patient care. It is noteworthy that there is little current research exploring how well clinical simulation can boost students’ self-confidence in Saudi Arabia. The results of this review may be used by educators, legislators and other interested parties to assess the relationship between clinical simulation and student confidence growth. The results of this review could also be applied to improve patients’ communication abilities. The ability to communicate with patients more confidently has been linked to clinical simulations. Students get a better understanding of patients’ needs once they feel comfortable speaking with them.

### Conclusion and recommendations

A total of 15 studies were retrieved for this literature-based study. The relevance of these studies ensured that the latest evidence on the use of clinical simulation in improving the self-confidence of student nurses was evaluated. Appropriate critiquing tools were utilised to evaluate each study. The findings of this review suggest that the self-confidence of student nurses is significantly improved following attendance in clinical simulation training in their undergraduate nursing curriculum. However, the research designs utilised in the studies included in this review act as a limitation to the applicability of the findings to a larger and more heterogeneous group.

### Contribution to the field statement

The inclusion of simulation-based learning in Saudi Arabia’s nursing curriculum may boost nursing students’ self-confidence. The present review, which is significant, demonstrated that simulation is substantially linked to improvements in self-confidence when speaking with patients and coworkers.

## Data Availability

The raw data supporting the conclusions of this article will be made available to the corresponding author (Nojoud Alrashidi) without undue reservation by the authors.
